# The combined effects of ultrasound and plasma-activated water on silkworm pupae:Physicochemical properties, microbiological diversity and ultrastructure

**DOI:** 10.1016/j.ultsonch.2024.106927

**Published:** 2024-05-24

**Authors:** Jia-Bao Ni, Shi-Ye Luo, Yan-Xiang Bi, Sara Zielinska, Chang-Jiang Ding, Jia-Li Tao, Zhen Ning, Wen-Li Tian, Wen-Jun Peng, Xiao-Ming Fang

**Affiliations:** a State Key Laboratory of Resource Insects, Institute of Apicultural Research, Chinese Academy of Agricultural Sciences, 1 Xiangshan Beigou, Beijing 100093, China; b College of Engineering, China Agricultural University, P.O. Box 194, 17 Qinghua Donglu, Beijing 100083, China; c Faculty of Mechanical and Power Engineering, Wroclaw University of Science and Technology, Wrocław, Poland; d College of Science, Inner Mongolia University of Technology, Hohhot, China

**Keywords:** Silkworm pupae, Ultrasound, Plasma activated water, Non-thermal processing, Antioxidant, Sterilization

## Abstract

A novel technique was proposed for processing silkworm pupae by combining plasma- activated water (PAW) with ultrasound (US). The microbial diversity and quality characteristics of the silkworm pupae were also evaluated. The results of the microbial diversity analysis indicated that PAW combined with US treatment significantly reduced the relative abundance of *Streptococcaceae*, *Leuconostocaceae*, and *Acetobacteraceae* from 32%, 18% and 16% to 27%, 11% and 11%, respectively. Microstructural analysis demonstrated that the collapse of the internal structure of chitin in silkworm pupae facilitated the release of nutrients and flavour compounds including fatty acids, water-soluble proteins (WSP), amino acids, phenolics, and volatile compounds. Furthermore, the increase in antioxidant capacity and the decrease in catalase activity and malondialdehyde content confirmed the mechanism of quality change. These findings provide new insights into the possible mechanism of PAW combined with US to improve the quality of edible insects.

## Highlights

• A new silkworm pupae processing technology for US combined PAW is proposed.
• US combined PAW significantly changed the microbial diversity and improved quality.
• RONS and cavitation effects are synergistically responsible for the quality change mechanism.
• Ultrastructural changes revealed the mechanism of nutrient release in silkworm pupae.

## Graphical abstract

**Figure:**
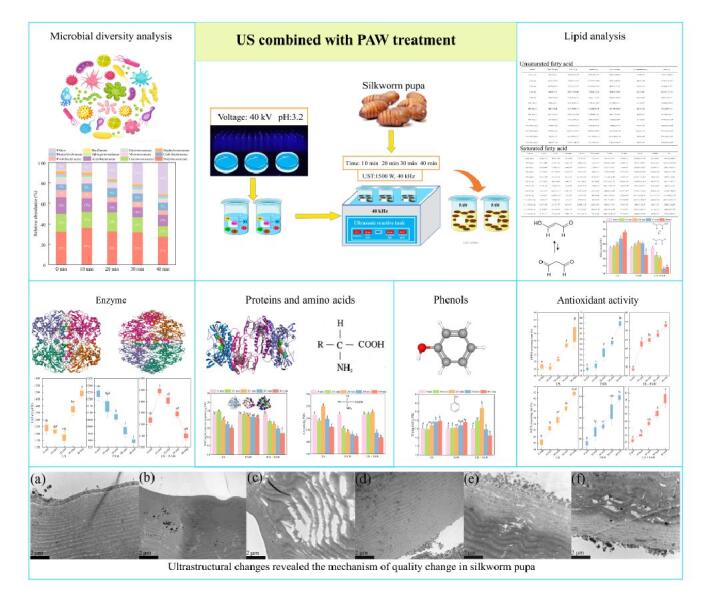

## 1 Introduction

According to the Food and Agriculture Organization of the United Nations (FAO), the global population is projected to exceed 9 billion by the middle of the 21st century. Current food production needs to increase by a minimum of 70 % to adequately sustain the global population [1]. However, factors such as COVID-19, the Russia-Ukraine conflict, global warming, water shortages, marine pollution, and overfishing significantly impacted on the global food supply chain, directly or indirectly leading to the chronic hunger of nearly 1 billion people worldwide, a number that continues to rise. Therefore, it is no longer sustainable to rely solely on expanding arable land or increasing the use of marine resources [1]. The promotion and exploration of alternative food sources with high yields, short life cycles, rapid reproduction, and environmental friendliness may prove to be effective solutions.

Silkworm pupae, rich in proteins, lipids, vitamins, polyphenols, peptides, essential amino acids, and trace elements, are among the most promising alternative animal food sources and have been consumed by humans for over 2,000 years. Silkworm pupae not only provide excellent nutrition but also exhibit anti-cancer, antioxidant, and immunomodulatory functions [2]. Globally, silkworm pupae production exceeds one million tons annually, with China contributing approximately 80 % of the world's output, playing a crucial role in feeding the billions of people in developing countries [2]. Despite this, silkworm pupae remain unpopular with consumers as a food item due to their high allergen content, microbial contamination, and unpleasant odour [1]. Researchers have explored thermal techniques, such as pasteurisation, hot air drying, and blanching, to inactivate spoilage enzymes and microorganisms and ensure the safety and quality of silkworm pupae products. However, applying heat, even at moderate temperatures, can have detrimental effects on flavour, essential nutrients (lipid oxidation and functional protein inactivation), and vitamins. Moreover, it may lead to the production of harmful amines, aldehydes, and glycosides, posing a serious threat to human health. For instance, He et al. reported that although heat treatment can reduce the sensitization of silkworm pupae, the tertiary structure of protein was completely destroyed by heat treatment at 80 °C for 5 min [3]. An investigation by Anuduang et al. revealed that prolonged exposure to hot water, coupled with high drying temperatures, could reduce the antioxidant activity of silkworm pupae [4]. Additionally, heat generated by resistance or combustion and transferred to the product requires relatively high energy, resulting in higher costs and being environmentally unfriendly.

Plasma-activated water (PAW) is a non-thermal processing technology with numerous advantages, gaining prominence as a research focal point. It is characterised by the storage of chemicals, including reactive oxygen species and reactive nitrogen species (RONS) (O_2_–, ·OH, H_2_O_2_, NO_3_–, and NO_2_–), generated by plasma gas–liquid discharge. These substances efficiently inactivate food-related pathogenic microorganisms and enzymes causing food quality deterioration (e.g., polyphenol oxidase, hydrogen peroxidase) at ambient temperature, thereby extending shelf life [5]. Moreover, PAW treatment has been shown to inhibit polyphenol oxidase in button mushrooms [6], and PAW exhibits antibacterial activity against *Salmonella enteritidis* and *Staphylococcus aureus* due to the presence of reactive nitrogen species [7]. However, it is crucial to note that improper use of PAW can result in excessive levels of nitrates (NO_3_–) and nitrites (NO_2_–) in food, potentially leading to the formation of carcinogenic N-nitroso compounds or their precursors, posing a public cancer risk [8]. Therefore, hurdle technology must be employed to ensure food safety, reduce the risk of residual nitroso compounds during PAW use, and fully harness the positive effects of PAW to enhance food quality and safety.

The distinctive non-thermal characteristics of US technology, such as the sponge effect and cavitation effect, set it apart among various processing technologies [9]. When ultrasonic waves penetrate materials containing a liquid medium, they induce a series of rapidly alternating compressions and expansions (the sponge effect). This effect creates microchannels inside microorganisms, leading to inner fluid leakage and bacterial death [10]. Simultaneously, cavitation bubbles generated by severe collapse during ultrasonication, along with free radicals produced by US and PAW (H_2_O_2_ and ∙OH), effectively interact with enzymes and deactivate them. To completely eliminate or inactivate harmful materials, a high-intensity working frequency is preferred, typically up to 16–100 kHz, causing rapid heating and pressurization of the liquid medium, disrupting cell membrane structures and resulting in lipid and protein modifications, thereby reducing food quality [11]. Studies indicate that an increase in ultrasonic intensity and processing time leads to elevated oxidation of beef proteins and lipids, along with the formation of harmful carbonyl compounds [12]. The combination of PAW and US has been already investigated in recent years. Thus to mitigate the negative quality impact of excessively high-intensity ultrasound work, the combination of PAW with US technology may be employed. US facilitates the deeper penetration of PAW into the food structure, allowing even a small amount of PAW to exhibit sterilizing and enzyme-inactivating effects while minimizing adverse impacts on food quality [13].

The objectives of this study were to assess: (1) the pretreatment efficiency of ultrasonication (US), plasma-activated water (PAW), and the combination of PAW with US in microbial inactivation of silkworm pupae; (2) the impact of US, PAW, and PAW combined with US on the quality parameters (such as antioxidant capacity, enzyme activity, total phenol content, and fatty acid content) and microbial diversity of silkworm pupae; and (3) the mechanism of PAW combined with US by observing and analysing the microstructure of silkworm pupae.

## 2 Materials and methods

### 2.1 Materials

Chemicals, including sodium chloride, sodium bicarbonate, and sodium hydroxide, were obtained from Xilong Scientific Co., Ltd (Guangzhou, China); methanol and hexane (HPLC-grade) were purchased from Fisher Chemicals (Fairlawn, NJ, U.S.A.); bovine serum protein standards, Coomas Bright Blue G-250 protein reagent, and FC reagent were acquired from Beijing Solarbio Science & Technology Co., Ltd (Beijing, China); gallic acid (GA) was obtained from Shanghai Yuanye Bio-Technology Co., Ltd (Shanghai, China); petroleum ether, phosphoric acid, and ethanol were purchased from Shanghai Aladdin Biochemical Technology Co., Ltd (Shanghai, China); sulfuric acid was obtained from Beijing Chemical Plant (Beijing, China). Silkworm pupae were sourced from the Institute of Sericology at the Chinese Academy of Agricultural Sciences, Zhenjiang City, Jiangsu Province, China, and stored in a refrigerator at −20 ± 1 °C until subsequent analysis. For the experiments, samples were selected to ensure consistency in physicochemical properties, with uniform dimensions of approximately 22–25 mm in length and 11–14 mm in width.

### 2.2 Preparation of PAW

**Fig. 1:**
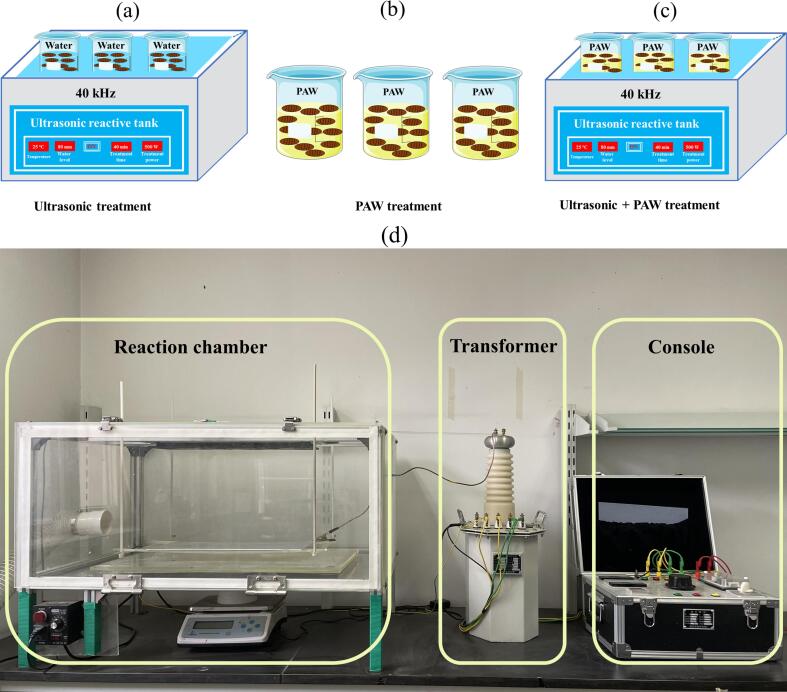
Silkworm pupae treated by PAW combined with US, US treatment (a), PAW treatment (b), PAW combined with US treatment (c). The dielectric barrier discharge system (d).

A non-thermal dielectric barrier discharge system was employed to generate PAW (Fig. 1). PAW generation involved a working gas of air present on the surface of ultrapure water, a treatment time of 4–5 h, an output voltage of 40 kV, a room temperature of 25 °C, and the spacing is 15 cm (Fig. 1). In a 150 mm diameter glass Petri dish, 300 mL of ultrapure water was added. After processing, the pH value, oxidation reduction potential and conductivity of PAW were 3.10 ± 0.20, 465.45 ± 6.15 mV, 449.50 ± 4.95 μs/cm, respectively.

### 2.3 Treatment of silkworm pupae

Fresh silkworm pupae served as the control group. In the US group, ultrapure water was added to silkworm pupae at a solid–liquid ratio of 1:10 and treated with US for 10, 20, 30, and 40 min at an ultrasonic frequency of 40 kHz and a power of 500 W. In the PAW group, silkworm pupae were treated with PAW at a solid–liquid ratio of 1:10 at room temperature for 10, 20, 30, and 40 min. For the PAW combined with US treatment group, PAW was added at a solid–liquid ratio of 1:10, and subsequently, the samples were exposed to US for 10, 20, 30, and 40 min at the same ultrasonic frequency of 40 kHz (Fig. 1). Following treatment, all samples were stored in sterile bags at −80 °C until further analysis. Each treatment was performed in triplicate to ensure the reliability and accuracy of the results.

### 2.4 Determination of fatty acid

Silkworm pupae fatty acids were extracted using the method outlined by Mohammad et al., with slight modifications [14]. Silkworm pupae (20 ± 1 g) were ground and mixed with 40 mL of saturated NaCl solution. After adding 250 mL of petroleum ether, the mixture underwent overnight oscillation and centrifugation (10 min, 10,000 rpm). The supernatant was collected, washed with ultrapure water, and the organic phase was freeze-dried. Subsequently, the extracted crude fat was methylated and analysed using an Agilent G3903-63011 GC–MS system equipped with a fused silica cyanopropyl DB-FastFAME column (30 mm × 0.25 mm, 0.25 μm, Agilent Tech.). Methyl-esterified fatty acids were identified by comparing retention times with standards, and quantification based on peak areas was performed using the NIST17 library.

### 2.5 Determination of water-soluble proteins content

The water-soluble protein content in silkworm pupae was determined following the method of Tang et al., with slight modifications [15]. 2 g of silkworm pupae were homogenised in 2 mL of ultrapure water in a mortar and transferred to a centrifuge tube. The mixture was then washed with 6 mL of ultrapure water, collected, and left for full extraction. After centrifugation (20 min at 4000 rpm), the volume was adjusted using ultrapure water. Colorimetric analysis was performed at 595 nm using Coomas Bright Blue G-250 protein reagent.

### 2.6 Determination of catalase activity, malondialdehyde content (MAD) and amino acid content

Catalase activity, malondialdehyde content, and amino acid content in the silkworm pupae were determined using a testing kit purchased from Beijing Solabo Biotechnology Co., Ltd, stored in a refrigerator at −4 °C.

### 2.7 Determination of total phenolic content

The total phenolic content of the silkworm pupae was determined using Ahmed et al.'s method with slight modifications [16]. Samples were dissolved in a mixture of methanol and water (3:1) to a concentration of 12.5 mg/mL. Subsequently, the mixture was shaken in a multi-tube vortex mixer for 15 min at 2500 rpm and centrifuged (5 min, 1000 rpm) in a Cence TGL-20 M centrifuge. Afterward, 20 µL of the supernatant was added to each well of a 96-well plate, followed by the addition of 100 µL of a 20 % aqueous sodium carbonate solution, 50 µL of diluted FC reagent (1:4), and 130 µL of ultrapure water. The plates were then incubated in the dark for 30 min at room temperature. Absorbance was measured at 765 nm using an enzyme-labelling apparatus. The total phenolic content was expressed as gallic acid equivalents (GAE). All treatments were conducted in triplicate.

### 2.8 Determination of antioxidant capacity

The antioxidant capacity of the silkworm pupae was determined following the method of Johnson Esua et al., with slight modifications [17]. Samples were prepared as previously described for the determination of total phenolic content. To assess the scavenging rate of DPPH radicals, the supernatant of the sample, a methanol–water solution (3:1, v/v), and a 0.2 mM DPPH radical solution were mixed and incubated for 15 min at room temperature. The absorbance was measured at 517 nm. For ABTS free radical scavenging rate, the supernatant of the sample was mixed with a solution of methanol–water (3:1, v/v) and a 0.2 mM ABTS radical solution, incubated at room temperature for 6 min, and then the absorbance was measured at 734 nm. The scavening rate is calculated by the following formula. All treatments were performed in triplicate.

$$\mathrm{Scavengingrate}=\left(1-\frac{A_{1}-A_{0}}{A_{2}}\right)\times 100\%$$

A_1_ is the absorbance of sample, A_0_ is the absorbance of control group, and A_2_ is the absorbance of blank group.

### 2.9 Determination of volatile compounds

Volatile compounds were analysed using an Agilent 5975C-7890A gas chromatography-mass spectrometer (Agilent, USA) with helium as the carrier gas (flow rate: 1.0 mL/min). Separation was performed on an HP-5MS UI chromatographic column (30 m × 0.25 mm × 0.25 μm, Agilent, USA). The program started with a column temperature of 50 °C for 5 min and then increased to 150 °C at a rate of 5 °C/min, with a hold of 5 min. Finally, the temperature increased at 5 °C/min to 250 °C with another hold for 5 min, and the program ended at 45 min in total. EI energy of 70 eV and a mass scan range of 29–450 *m*/*z* were used. For preliminary identification, the NIST library and G.A.S GC-IMS database were used, with matches exceeding 80 % deemed acceptable. The relative content of the volatile compounds was determined by calculating the peak area ratio of each compound to that of the blank control under each condition.

### 2.10 Microstructure by transmission electron microscopy (TEM)

TEM was employed to examine the microstructure of the silkworm pupae epidermis. Initially, epidermis specimens were fixed in a 0.1 M phosphate buffer with a pH of 7.2, containing 2.5 % glutaraldehyde. Subsequently, further fixation occurred in a phosphate buffer with 1 % osmium tetroxide for 2 h. Afterward, the samples underwent triple 10 min. washes. Following this, dehydration was achieved through a series of ethanol solutions with varying concentrations (35 %, 50 %, 70 %, 90 %, 95 %, and 100 %). The samples were left overnight at 4 °C in a mixture of propylene oxide and Spurr embedding resin, followed by embedding in pure resin. Thin sections were obtained using an ultramicrotome (Leica EM UC6; Wetzlar, Germany) and collected on copper grids. Staining with uranyl acetate and lead citrate was performed, and the sections were examined under a Hitachi H-7560 transmission electron microscope at 12,000x magnification (Hitachi Hi-tech, Tokyo, Japan).

### 2.11 Analysis of microbial composition

The microbial diversity of silkworm pupae subjected to PAW combined with US treatment at different time intervals was analysed using high-throughput sequencing. DNA extraction from silkworm pupae followed the instructions of test kit, with PCR amplification of the variable region of the rRNA gene or specific gene fragments. Sequencing libraries were prepared using the TruSeq Nano DNA LT Library Prep Kit (Illumina, CA, USA). Sequencing data were spliced and filtered to eliminate chimeras, discarding low-quality sequences to obtain high-quality sequences for accurate analysis. These high-quality sequences were used for Operational Taxonomic Unit (OTU) classification, diversity index calculations, and species abundance analyses.

### 2.12 Statistical analysis

All experiments were conducted in triplicate. Significance levels were set at P < 0.05, and the variables were analysed using multivariate analysis of variance (MANOVA). IBM SPSS Statistics 22.0 and Origin 2023 were employed for all analyses and figure generation.

## 3 Results and discussion

### 3.1 Effect of PAW combined with US on the fatty acids of silkworm pupae

Silkworm pupae are significant contributors to essential fatty acids, especially unsaturated fatty acids. These acids play a vital role in lowering lipid levels, preventing weight gain, and addressing obesity. Major fatty acids in silkworm pupae crude fat include oleic acid, linoleic acid, α-linolenic acid, palmitic acid, and stearic acid (Table 1). Following 40 min of US treatment, there was a significant increase *(P <* 0.05*)* in total unsaturated fatty acids (211106.76 ± 1260.44 mg/kg DWT), encompassing α-Linolenic (102303.56 ± 930.16 mg/kg DWT), linoleic (29222.11 ± 592.28 mg/kg DWT), and oleic (73628.29 ± 325.81 mg/kg DWT), increased by 13.46 %, 63.49 % and 23.64 % respectively compared with the control group. Similarly, saturated fatty acids (58031.03 ± 35.89 mg/kg DWT), including palmitic (33131.44 ± 291.08 mg/kg DWT) and stearic (21610.31 ± 272.43 mg/kg DWT), also significant increased by 49.45 % and 17.79 % respectively compared with the control group *(P <* 0.05*)*. US treatment enhanced the total fatty acid content in silkworm pupae crude fat through mechanisms such as cavitation, erosion, sonocapillary effects, and sonoporation. Cavitation involves the formation and collapse of microscopic bubbles, leading to increased mass transfer, disruption of cell walls, and the induction of temporary pores in the cell membrane, facilitating solvent flow and extraction [10]. Studies have shown that US-assisted extraction increases the fatty acid content of flaxseed oil, particularly polyunsaturated fatty acids [18]. Interestingly, prolonged exposure to PAW alone reduced unsaturated fatty acids, it was decreased by 2.07 % and 9.06 % compared with the control after treatment for 30 min and 40 min, while it was increased by 8.93 %, 31.51 %, 36.52 % and 12.14 % respectively compared with the control group after combined application of PAW and US for 10 min, 20 min, 30 min and 40 min. In contrast, the content of saturated fatty acids significantly increased with the duration of the combined treatment, it was increased by 2.95 %, 51.91 %, 54.01 % and 73.15 % respectively compared with the control group after combined application of PAW and US for 10 min, 20 min, 30 min and 40 min and reaching 74165.10 ± 993.00 mg/kg DWT after 40 min *(P <* 0.05*)*. In the process of fatty acid oxidation, reactive species such as RONS in PAW play a significant role in removing two hydrogen atoms from the fatty acid molecule, resulting in the production of a double bond between the alpha and beta carbons susceptible to oxidation [19]. The sonocapillary effect of US generates capillary waves on the PAW, promoting contact with the silkworm pupae and enhancing fatty acid oxidation during prolonged PAW combined with US treatment, particularly for oleic and α-Linolenic acids. However, unsaturated fatty acids are more prone to oxidation due to the presence of double bonds, resulting in reduced unsaturated fatty acid content during PAW and PAW combined with US treatment. In contrast, saturated fatty acid content continues to increase as it is chemically stable and does not easily react with other molecules or break down. PAW has been shown to induce fatty acid oxidation in grass carp [8], while Astorga et al. found that PAW treatment significantly reduced lipid oxidation in beef [20]. This indicates that NO_2_– functions as an antioxidant in meat products through chemical mechanisms, including forming a strong complex with heme pigments, preventing the release of non-haeme iron, and inhibiting its catalytic effect on lipid oxidation. Additionally, NO_2_– interacts with free non-haeme iron, contributing to the stabilization of unsaturated lipids within the cell membrane [20]. An appropriate combination of US and PAW treatments holds promise for enhancing the quality of silkworm pupae by improving fatty acid content and reducing the oxidation of unsaturated fatty acids.

**Table 1:** The total fatty acid composition of silkworm pupae crude fat under different treatment conditions. Different letters (a–e) show statistically significant difference *(P* < 0.05*)*.

Fatty acid (mg/kg)	CK	US (10 min)	US (20 min)	US (30 min)	US (40 min)	PAW (10 min)	PAW (20 min)	PAW (30 min)	PAW (40 min)	US + PAW (10 min)	US + PAW (20 min)	US + PAW (30 min)	US + PAW (40 min)
*Saturated fatty acid*													
Capric	20.39 ± 0.99^ef^	29.07 ± 0.80^b^	30.14 ± 0.68^b^	28.45 ± 0.84^bc^	30.98 ± 1.09^b^	18.73 ± 3.39^f^	16.70 ± 1.44^f^	23.45 ± 2.71^de^	25.26 ± 2.38 ^cd^	24.96 ± 3.27 ^cd^	29.29 ± 1.00^b^	30.56 ± 0.57^b^	40.34 ± 1.11^a^
Lauric	289.20 ± 3.95^fg^	297.57 ± 1.15^f^	294.28 ± 0.92^f^	328.45 ± 9.82^d^	343.62 ± 3.61^c^	303.05 ± 9.06^ef^	315.43 ± 5.04^de^	263.90 ± 11.31^hi^	276.22 ± 11.32^gh^	259.56 ± 7.20^i^	369.36 ± 7.78^b^	360.05 ± 4.68^b^	391.64 ± 9.31^a^
Tridecanoic	11.80 ± 0.50^abcd^	12.62 ± 2.21^abc^	13.66 ± 1.60^abc^	11.24 ± 1.59^bcd^	11.10 ± 2.10^cd^	8.08 ± 1.58^ef^	14.31 ± 2.28^a^	12.78 ± 0.71^abc^	9.50 ± 1.23^de^	6.20 ± 1.19^f^	14.19 ± 0.55^ab^	12.74 ± 0.41^abc^	13.98 ± 1.44^abc^
Myristic	772.96 ± 7.53^h^	1098.05 ± 22.42^f^	1216.28 ± 18.00^e^	1333.12 ± 13.00^d^	1413.36 ± 10.42^cd^	888.24 ± 32.08^g^	813.52 ± 25.73^gh^	871.09 ± 124.00^gh^	874.37 ± 9.10^gh^	1057.85 ± 82.61^f^	1505.08 ± 33.38^bc^	1595.70 ± 12.14^b^	1853.74 ± 103.47^a^
Pentadecanoic	262.23 ± 4.36^fg^	273.23 ± 9.85^ef^	273.89 ± 4.05^ef^	304.40 ± 2.13^d^	316.08 ± 7.00^cd^	268.47 ± 8.64^f^	275.06 ± 8.26^ef^	210.11 ± 10.57 ^h^	286.97 ± 8.58^e^	246.77 ± 3.93 ^g^	325.46 ± 12.07^c^	358.27 ± 11.49^b^	376.01 ± 3.98^a^
Palmitic	22169.10 ± 181.32^j^	25529.74 ± 479.06^g^	27136.67 ± 194.57^f^	31570.71 ± 570.94^e^	33131.44 ± 291.08^d^	23967.55 ± 572.15^hi^	24438.62 ± 592.58^gh^	22846.25 ± 144.02^ij^	22440.70 ± 410.38^j^	21892.59 ± 1547.66^j^	36300.67 ± 477.21^c^	38591.60 ± 301.64^b^	40564.59 ± 1031.64^a^
Margaric	501.02 ± 3.02^i^	543.56 ± 6.56^f^	544.64 ± 3.41^f^	593.53 ± 5.95^e^	620.48 ± 7.67^d^	535.76 ± 6.73 ^fg^	513.86 ± 12.58^hi^	517.50 ± 19.87^ghi^	512.23 ± 9.63^hi^	528.78 ± 7.89^fgh^	690.50 ± 7.14^c^	734.42 ± 7.24^a^	712.29 ± 9.80^b^
Stearic	18346.85 ± 959.12^fgh^	18053.81 ± 938.95^gh^	20544.52 ± 538.61^def^	20549.70 ± 829.18^def^	21610.31 ± 272.43^cde^	20243.42 ± 221.05^efg^	22594.92 ± 625.10^cd^	17919.52 ± 1742.76^h^	21075.36 ± 1861.88^de^	19631.32 ± 1286.43^efgh^	25206.65 ± 212.14^b^	23730.95 ± 542.00^bc^	29564.43 ± 1955.63^a^
Arachidic	458.32 ± 8.49^ef^	450.28 ± 5.63^ef^	487.59 ± 9.54^d^	544.53 ± 10.59^c^	553.66 ± 8.59^c^	446.25 ± 5.77^f^	459.83 ± 4.48^ef^	465.26 ± 13.02^e^	456.47 ± 11.79^ef^	446.67 ± 12.16^f^	623.63 ± 5.58^b^	551.14 ± 5.36^c^	648.07 ± 6.01^a^
Total amount	42831.87 ± 1040.17^h^	46287.93 ± 1408.69^fg^	50541.67 ± 325.67^e^	55264.12 ± 1219.81^d^	58031.03 ± 35.89^c^	46679.54 ± 727.05^f^	49442.24 ± 16.10^e^	43129.88 ± 1903.18^h^	45957.08 ± 2214.81^fg^	44094.71 ± 1856.43^gh^	65064.82 ± 722.58^b^	65965.42 ± 247.34^b^	74165.10 ± 993.00^a^

*Unsaturated fatty acid*													
Palmitoleic	3359.71 ± 61.51^e^	4541.50 ± 316.57^c^	4857.78 ± 155.01^b^	5045.08 ± 53.15^b^	5520.50 ± 95.29^a^	3240.85 ± 102.71^e^	2924.65 ± 45.28^f^	2830.73 ± 51.12 ^fg^	2644.74 ± 19.23 ^g^	4357.86 ± 137.26^c^	5773.50 ± 141.45^a^	4559.76 ± 94.54^c^	4082.31 ± 144.26^d^
Oleic	59549.52 ± 662.12^gh^	57404.52 ± 406.15^i^	62131.22 ± 326.97^f^	69547.19 ± 999.58^de^	73628.29 ± 325.81^c^	60298.58 ± 936.72 ^g^	59735.66 ± 491.54^g^	57947.79 ± 271.84^hi^	54557.52 ± 1292.21^e^	67971.78 ± 666.91^j^	80159.71 ± 1253.55^b^	83409.78 ± 319.63^a^	70484.17 ± 1308.39^d^
Linoleic	17874.47 ± 632.57 ^g^	21337.77 ± 1349.49^f^	25590.49 ± 1007.95^d^	27256.45 ± 225.29^c^	29222.11 ± 592.28^b^	17139.53 ± 172.06^gh^	17429.02 ± 184.29^g^	15960.00 ± 65.07 ^h^	13643.58 ± 104.41^i^	23830.36 ± 387.04^e^	30573.10 ± 453.42^ab^	31114.25 ± 1194.15^a^	25645.53 ± 872.13^d^
α-Linolenic	90148.95 ± 1085.65^hi^	95260.33 ± 837.39 ^fg^	98040.94 ± 173.21^ef^	99354.82 ± 974.43^de^	102303.56 ± 930.16 ^cd^	103179.80 ± 3135.11^c^	93511.09 ± 182.81^gh^	90695.53 ± 413.29^hi^	84630.43 ± 200.97^j^	90090.68 ± 771.57^i^	108310.72 ± 1423.05^b^	114321.58 ± 4282.56^a^	91458.15 ± 1056.78^hi^
Eicosapentaenoic	374.34 ± 4.07^ef^	384.31 ± 3.98^e^	397.62 ± 5.16^d^	421.38 ± 8.91^c^	432.30 ± 6.93^c^	372.08 ± 3.73^f^	322.53 ± 4.25^h^	320.43 ± 6.17^h^	303.00 ± 5.28^i^	357.15 ± 4.75 ^g^	474.40 ± 11.16^a^	459.12 ± 2.84^b^	433.39 ± 4.24^c^
Total amount	171306.97 ± 108.37^hi^	178928.42 ± 664.25^g^	191018.05 ± 800.75^e^	201624.93 ± 1725.03^d^	211106.76 ± 1260.44^c^	184230.84 ± 4086.75^f^	173922.95 ± 493.23^h^	167754.48 ± 313.61^i^	155779.26 ± 1173.12^j^	186607.84 ± 1561.03^f^	225291.44 ± 2998.60^b^	233864.50 ± 3674.83^a^	192103.56 ± 1139.80^e^

### 3.2 Effect of PAW combined with US on the WSP of silkworm pupae

**Fig. 2:**
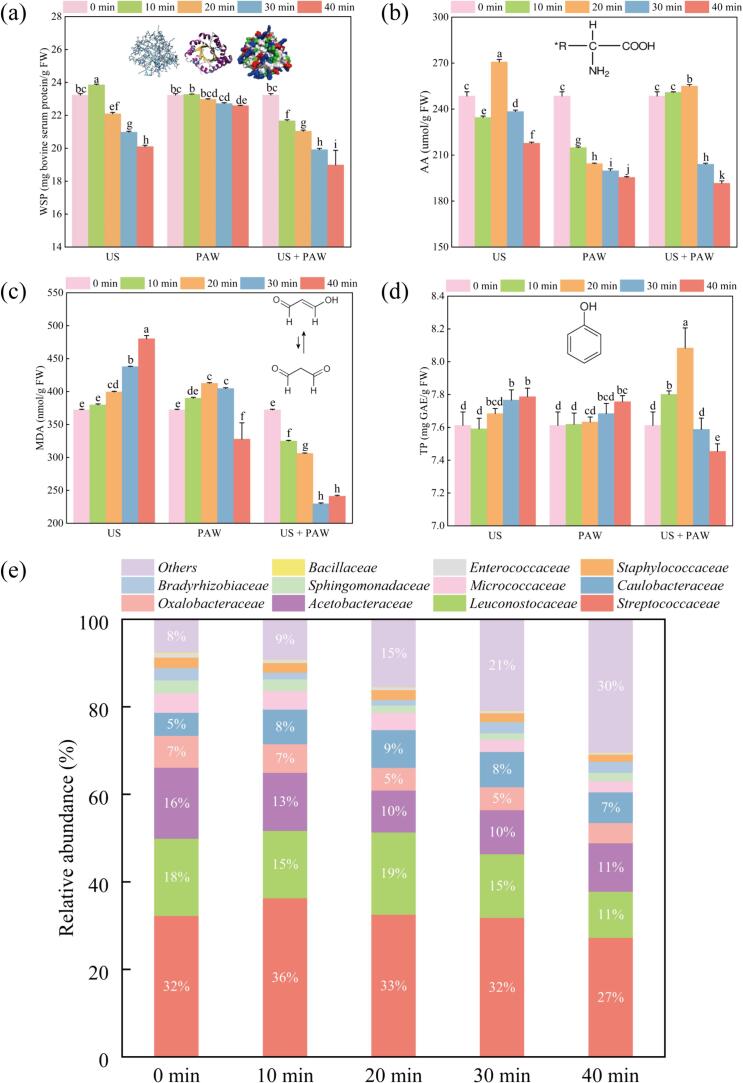
The water-soluble proteins content (a), amino acid content (b), malondialdehyde content (c), total phenol content (d) of silkworm pupae under US, PAW and PAW combined with US treatment, and microbial diversity of silkworm pupae under PAW combined with US treatment at different time (e). Different letters (a-i) show statistically significant difference *(P* < 0.05*)*.

The excellent solubility of WSP in food is highly desirable due to its crucial role in foaming, emulsification, and gel characteristics. Fig. 2 (a) illustrates the WSP content of silkworm pupae under various treatment conditions. In the US group, there was an initial increase reaching a peak of 23.86 ± 0.05 mg/g FW after 10 min, followed by a subsequent decrease to 20.10 ± 0.09 mg/g FW at 40 min *(P <* 0.05*)*. US treatment induces chemical changes in proteins, leading to conformational alterations and modifications of amino acid side groups. Simultaneously, enhanced molecular agitation from US cavitation can induce temporary or permanent modifications to the folded protein structure [9]. Consequently, US treatment can improve protein solubility by reducing protein size and breaking insoluble aggregates. However, excessive treatment can result in denaturation and aggregation, leading to reduced solubility and functionality [15], [21]. Moreover, Kang et al. confirmed that US treatment can enhance protein solubility under appropriate conditions; however, excessive treatment may lead to decreased solubility [22]. Notably, WSP content significantly dropped to 22.59 ± 0.03 mg/g after 40 min of PAW treatment *(P <* 0.05*).* The combination of PAW with US treatment resulted in a more significant decrease to 18.98 ± 0.90 mg/g after 40 min *(P* < 0.05*)*, demonstrating its more pronounced effectiveness compared to individual treatments. The reduction in WSP levels in silkworm pupae subjected to PAW treatment can be ascribed to the presence of active species, including RONS, which can interact with proteins and modify their structure and function [17]. The intensified reduction observed in the combined treatment may be linked to the potentiated oxidation process and the generation of hydroxyl radicals through the additive cavitation effects of US [13]. For example, prolonged PAW treatment decreased the solubility of proteins from *Aristichthys* nobilis myofibrillar protein by inducing the transformation from α-helix and random coil to β-sheet and β-turn due to PAW containing a large amount of O_3_, NO_2_^–^, NO_3_^–^ and a small amount of H_2_O_2_ [23]. Johnson Esua et al. reported that the combination of US and PAW disrupted intermolecular hydrophobic and electrostatic interactions between protein molecules in small yellow croaker, leading to reduced protein content [17]. However, the variations in the results among these studies can be attributed to differences in processing parameters, raw materials, and operation methods.

### 3.3 Effect of PAW combined with US on the amino acids of silkworm pupae

Silkworm pupae contain nine essential amino acids essential for normal human growth and development [1]. In Fig. 2(b), the amino acid content increased to 270.74 ± 1.68 μmol/g FW after 20 min, then significantly decreased to 217.75 ± 0.72 μmol/g FW after 40 min *(P <* 0.05*)* compared to the control in the US group. The mechanical effects of US promote amino acid release by disrupting the microstructure and increasing the activity of proteolytic enzymes. Meanwhile the chemical effects involve chain breakage and modification of amino acid side groups [9]. Moreover, abusive US treatment leads to enzyme inactivation and protein denaturation, ultimately causing amino acid destruction [22]. Nevertheless, the effect of US treatment on amino acid content varies depending on the protein source. For example, US treatment increased the total amino acid content of mung bean protein hydrolysates [24]. Conversely, in spiced beef, US treatment reduces the total amino acid content, particularly glutamic and aspartic acids, while increasing the content of other free amino acids [12]. Our results showed that the total amino acid content continuously decreased to 195.44 ± 0.48 μmol/g FW after 40 min of PAW treatment. Despite the slight increase in amino acid content, the combined US and PAW treatment resulted in a significant decrease to 191.60 ± 1.57 μmol/g FW after 40 min *(P* < 0.05*)*. The decrease in amino acid content caused by PAW is attributed to reactive species such as RONS, inducing chemical modifications in amino acids through hydroxylation, nitration, dehydrogenation, and dimerization [5]. Additionally, the combined application of US and PAW led to a synergistic effect on amino acids, causing a notable reduction in their quantity, suggesting that US potentially aids in the interaction between PAW and the amino acids present in silkworm pupae. Although limited information exists on the synergistic effect of PAW treatment on amino acids, research indicates that PAW treatment significantly reduces tryptophan and tyrosine concentrations, with cold plasma mode-dependent hydroxylation and nitration of aromatic rings in these amino acids [25]. To date, there is limited evidence regarding the effects of PAW and US treatments on the amino acid content of silkworm pupae. Further research is necessary to understand the specific mechanisms of combining these treatments.

### 3.4 Effect of PAW combined with US on the MDA of silkworm pupae

Malondialdehyde (MDA) is a byproduct of lipid peroxidation, and its production reflects the extent of lipid oxidation. As depicted in Fig. 2(c), the MDA content in silkworm pupae increased significantly with prolonged treatment time, reaching its peak value (480.21 ± 4.85 nmol/g FW) at 40 min in the US treatment. This value was significantly higher than the MDA content in the control group (372.26 ± 0.97 nmol/g FW) *(P* < 0.05*)*. The mechanism underlying ultrasound-induced MDA production by US is primarily associated with lipid oxidation. US generates high-temperature and high-pressure micromechanical shock waves, known as cavitation, along with highly active free radicals that impact the structural and functional components of lipids, causing their oxidation and deterioration. Meanwhile, the US cavitation zone, characterised by higher lipid oxidation due to elevated temperature and the presence of free radicals, produces numerous free radicals (e.g., hydroxyl, hydrogen, and perhydroxyl radicals) through US, leading to the decomposition and oxidation of polyunsaturated fatty acids and the formation of a peroxidation byproduct (MDA) [26]. Subsequent studies have confirmed a positive correlation between MDA content and ultrasound power and time. Bao et al. observed an increase in MDA content with rising US power and time during the ultrasound-assisted thawing of frozen white yak meat [27]. In the treatment with PAW, the MDA content initially increased to 412.47 ± 1.13 nmol/g FW after 20 min, then significantly decreased to 327.74 ± 24.86 nmol/g FW after 40 min (*P* < 0.05). This decrease is attributed to the ability of RONS produced in PAW to interact directly with lipids. It is characterised by the abstraction of hydrogen atoms from the fatty acid chain, initiating lipid oxidation, particularly in unsaturated fatty acids with unsaturated bond characteristics (Table 1) [19]. Lotfy et al. (2022) reported that PAW treatment increased the levels of lipid oxidation products such as MDA [28], consistent with our results. Notably, a significant decrease in MDA content was observed as treatment time increased, reaching its lowest value (229.57 ± 1.45 nmol/g FW) at 30 min in the combined US and PAW treatment. Our findings indicate that the combined treatment significantly reduced MDA accumulation. Furthermore, the synergistic effect of combined US and PAW treatment inhibited MDA formation. This can be attributed to the role of US treatment in facilitating the penetration of PAW into the substance via acoustic cavitation. This promotes the reaction of active particles with other oxidising substances, including MDA, neutralising them under acidic conditions [29], thus significantly reducing MDA content in silkworm pupae.

### 3.5 Effect of PAW combined with US on the total phenol of silkworm pupae

Phenolic compounds in food exhibit remarkable antioxidant capacities, effectively scavenging free radicals and serving as potent electron donors. In Fig. 2 (d), the total phenolic content in silkworm pupae increased with prolonged US treatment, peaking at 7.79 ± 0.05 mg GAE/g FW after 40 min, significantly higher than the control *(P* < 0.05*)*. This can be attributed to the mechanical impact of US, which breaks down particles, causing peeling, erosion, and disruption of cellular structures. Consequently, bound phenolic compounds are liberated, and the solubility of phenolic compounds increases through the breakdown of complexes with other molecules such as proteins. Studies have reported increased total phenolic content in *Commiphora gileadensis* following US treatment due to sonication-generated acoustic cavitation bubbles, which increase the extraction temperature and destroy the plant cell wall. Consequently, more phenolic compounds are released into the solution matrix [16]. Similarly, the PAW treatment demonstrated a similar trend, reaching a total phenolic content of 7.76 ± 0.04 mg GAE/g FW after 40 min. This is because PAW not only prompts RONS generation to activate oxidative stress defence mechanisms but also stimulates enzyme activity and gene expression linked to polyphenol metabolism, ultimately increasing the synthesis or release of phenolic compounds [5]. However, PAW potentially reacts with phenolic compounds, leading to their oxidation and reduced phenolic content. Therefore, in the combined treatment, the total phenol content significantly surged to 8.08 ± 0.12 mg GAE/g FW *(P* < 0.05*)* after 20 min and then decreased to 7.45 ± 0.05 mg GAE/g FW after 40 min *(P* < 0.05*)*. Additionally, studies have demonstrated that excessive RONS in PAW lead to oxidative damage, accelerating the degradation of phenolic compounds in fresh-cut pears [30]. In silkworm pupae, the combined US and PAW treatment synergistically increased the total phenolic content, suggesting its potential application in enhancing antioxidant functionality.

### 3.6 Effect of PAW combined with US on the antioxidant activity of silkworm pupae

**Fig. 3:**
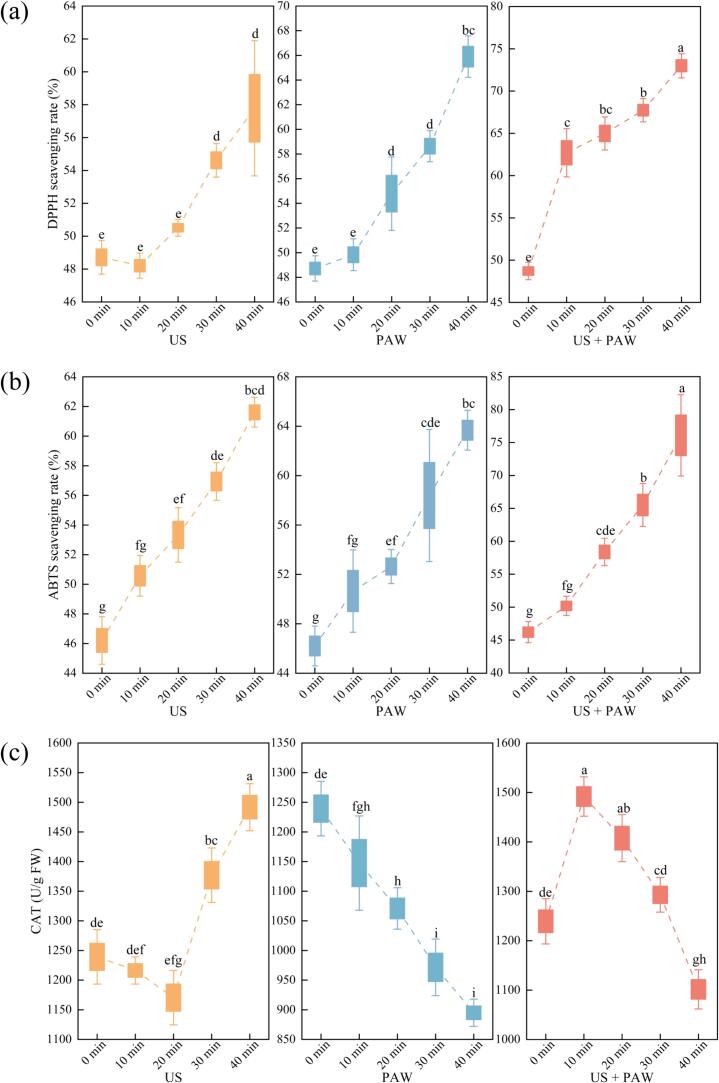
The DPPH (a) and ABTS (b) scavenging rate, and catalase activity (c) of silkworm pupae under US, PAW and PAW combined with US treatment. Different letters (a-i) show statistically significant difference (*P* < 0.05).

Silkworm pupae exhibit remarkable antioxidant capacity, attributed to a diverse range of bioactive compounds, including unsaturated fatty acids, peptides, amino acids, and phenolic compounds. The antioxidant capacity was assessed using DPPH and ABTS radical-scavenging assays (Fig. 3 (a, b)). Treatment with US, PAW, or their combination significantly increased the antioxidant capacity of silkworm pupae. After 40 min of US treatment, compared to the control, the silkworm pupae demonstrated a significant increase in the scavenging rate of DPPH and ABTS radicals, reaching 57.78 ± 4.11 % and 61.61 ± 1.00 %, respectively *(P <* 0.05*)*. As widely recognised, the enhanced antioxidant capacity resulting from US treatment can be attributed to its physical and chemical effects on the microstructure and nutritional components of silkworm pupae, including proteins, peptides, amino acids, antioxidants, and phenolic compounds. US cavitation not only promotes protein unfolding, exposing more active antioxidant sites, but also increases the content of aromatic and hydrophobic amino acids, serving as electron donors to scavenge free radicals [26]. Additionally, US treatment boosts the activity of antioxidant enzymes, such as superoxide dismutase and catalase, through the production of reactive free radicals [31]. The application of PAW significantly improved the antioxidant capacity of silkworm pupae. The scavenging rates were higher than the control group by 17.18 % and 17.47 % for DPPH and ABTS, respectively, after 40 min of treatment. Similarly, this improvement is attributed to the generation of RONS, inducing oxidative stress and upregulating antioxidant enzyme activity, including catalase, thereby increasing the antioxidant capacity. Moreover, RONS stimulate the production of secondary metabolites, such as phenolic acids, known for their antioxidant properties [5]. Numerous studies support the efficacy of US and PAW treatments in enhancing the antioxidant capacity of various food products. For instance, US treatment increases the antioxidant activity of mung bean protein hydrolysate [24], while PAW treatment improves the antioxidant capacity of strawberries and positively affects their phenolic profile, vitamin content, and antioxidant activities of rocket-salad leaves [32]. Remarkably, the combined treatment demonstrated the most potent effects, yielding scavenging rates of 73.00 ± 1.43 % (DPPH) and 76.09 ± 6.17 % (ABTS) after 40 min, significantly higher than the control and other treatments *(P* < 0.05*)*. The combined US and PAW treatment has a well-known synergistic effect on enhancing the antioxidant capacity of silkworm pupae, owing to the generation of acoustic cavitation, micromechanical shockwaves, and microjets, facilitating mass transfer and PAW penetration [17]. Consequently, PAW combined with US treatment has shown promise in generating more radical-scavenging fragments and enhancing antioxidant capacity.

### 3.7 Effect of PAW combined with US on the catalase activity of silkworm pupae

Catalase, a crucial enzyme involved in oxidative stress and microbial contamination, was investigated in silkworm pupae subjected to various treatments (Fig. 3 (c)). After 40 min of US treatment, catalase activity significantly increased to 1491.75 ± 39.75 U/g FW, compared to the control group (1239.30 ± 45.90 U/g FW) *(P* < 0.05*)*. This increase can be attributed to the ability of US to disrupt cell membranes, allowing the enzyme to be released and become more accessible. Additionally, US treatment can enhance enzyme activity by inducing alterations in the molecular structure, such as β-sheet, random coil, and β-antiparallel, rendering the catalytic centre more conducive to substrate catalysis [33]. Further investigations into β-glucosidase enzyme activity consistently revealed the potent positive impact of US treatment [34]. In contrast, PAW treatment reduced catalase activity to 895.05 ± 22.95 U/g FW *(P* < 0.05*)* after 40 min. Uniquely, PAW treatment affects catalase activity through the presence of RONS generated during the plasma activation process, and the pH of the PAW used in the present study (pH 3.3 ± 0.2) falls outside the optimal pH range for catalase activity (pH 7 to pH 11). Both factors may hinder the enzyme's function [17]. Similarly, PAW treatment has been shown to inhibit polyphenol oxidase activity in button mushrooms [6]. However, RONS, such as hydrogen peroxide, can trigger oxidative stress in cells, prompting the upregulation of antioxidant enzyme activity as a protective mechanism against cellular damage [5]. The duration of this defence mechanism may vary depending on the organism and the type of stress encountered. This variation may explain the observation that the combined US and PAW treatment initially increased catalase activity to 1407.60 ± 47.77 FW U/g after 20 min, and then decreased to 1101.60 ± 39.75 FW U/g *(P <* 0.05*)* after 30 min, a level significantly lower than the control. Moreover, Soni et al. reported the deeper penetration of reactive species into the cells and the induction of oxidative stress on bacterial cell membranes following the combined US and PAW treatment, thereby activating defence mechanisms [35]. This is consistent with our results, suggesting that the combined treatment might induce oxidative stress defence mechanisms in silkworm pupae, ultimately leading to the upregulation of catalase release.

### 3.8 Effect of PAW combined with US on the volatile compounds of silkworm pupae

A total of 36 volatile components were detected in the silkworm pupae, comprising 28 alkanes, 5 esters, and 3 aromatic components. The relative content of each volatile component was calculated using the ion peak area of the control as the denominator (Table 2). In the groups treated with US or PAW combined with US, there was a significant decrease in the levels of alkanes and phenols. This reduction is primarily attributed to the mechanical damage inflicted on cells and tissues by US, resulting in the release of volatile compounds alongside heightened thermal effects. Consequently, there is a loss of volatile components such as alkanes and aromatics in silkworm pupae [12]. Furthermore, US treatment promoted lipid oxidation and the release of fatty acids, facilitating ester production (Table 1). Consequently, after 40 min of US treatment, the relative content of total esters increased significantly to 129.50 ± 0.83 % compared to the control *(P* < 0.05*)*. However, the ester content in silkworm pupae decreased significantly after PAW treatment or PAW combined with US treatment. This decline is primarily attributed to the acidity of PAW, which hydrolyses esters [36]. Notably, benzenes and esters of short-chain acids contribute to the fruity flavour. In this study, it was observed that tributyl phosphate decreased to 40.55 ± 0.91 % of the control after 40 min of PAW combined with US treatment *(P* < 0.05*)*. This compound mildly inhibits cholinesterase in human plasma and may cause poisoning [37]. Additionally, alkanes are products of fat oxidation, where n-alkanes may arise from the auto-oxidation of fat, and branched alkanes could originate from the oxidation of branched fatty acids in raw materials [36]. Although the overall alkane content in the PAW treatment group was lower than that in the control, it is interesting to note that both PAW treatment and the appropriate combination of PAW with US significantly increased the relative content of alkanes. The peak values were observed as 91.15 ± 1.40 % after 40 min of PAW treatment and 125.18 ± 3.97 % after 10 min of PAW combined with US treatment. Nevertheless, this strongly supports the notion that RONS in PAW are prone to lipid oxidation, and an appropriate combination of treatments may promote the release of flavour substances while degrading harmful components.

**Table 2:** Volatile compounds in the silkworm pupae under different treatment conditions are analysed. Different letters (a–e) indicate statistically significant differences *(P* < 0.05*)*.

Volatile compounds (Relative abundance %)	US (10 min)	US (20 min)	US (30 min)	US (40 min)	PAW (10 min)	PAW (20 min)	PAW (30 min)	PAW (40 min)	US + PAW (10 min)	US + PAW (20 min)	US + PAW (30 min)	US + PAW (40 min)
*Alkane*												
N-octane	111.97 ± 1.8^a^	95.69 ± 6.02^b^	68.22 ± 3.96^de^	60.61 ± 0.98^def^	60.41 ± 7.49^def^	71.78 ± 4.66 ^cd^	60.63 ± 1.92^def^	50.12 ± 6.88^fg^	81.07 ± 11.29^c^	54.72 ± 0.46^fg^	58.74 ± 2.19^ef^	43.32 ± 2^g^
Heptane, 2,4-dimethyl-	53.65 ± 1.79^ef^	27.74 ± 4.01^g^	68.81 ± 10.24 ^cd^	30.56 ± 8.88^g^	73.76 ± 1.2^c^	94.86 ± 5.88^b^	80.9 ± 0.89^c^	60.56 ± 5.93^de^	132.12 ± 7.08^a^	60.05 ± 2.67^de^	60.03 ± 2.42^de^	46.51 ± 0.36^f^
Octane, 4-methyl-	57.78 ± 0.77^f^	33.69 ± 1.12^h^	52.8 ± 1.61^f^	42.26 ± 2^g^	66.53 ± 1.04^e^	88.34 ± 0^c^	79.17 ± 3.32^d^	68.49 ± 1.21^e^	129.62 ± 7.72^a^	66.17 ± 2.23^e^	65.77 ± 3.28^e^	53.76 ± 2.16^f^
N-nonane	101.25 ± 4.6^a^	63.39 ± 0.07^c^	53.69 ± 2.75^d^	42.26 ± 4.18^e^	59.4 ± 4.46^cd^	63.97 ± 2.45^c^	61.92 ± 0.98^c^	58.79 ± 5.11^cd^	76.61 ± 2.97^b^	62.05 ± 1.24^c^	53.39 ± 1.39^d^	54.35 ± 1.58^d^
Nonane, 4-methyl-	109.05 ± 2.01^b^	48.24 ± 0.66^h^	62.99 ± 0.97 ^g^	52.71 ± 3.75^h^	74.68 ± 1.67^f^	87.72 ± 0.98^d^	98.73 ± 2.58^c^	72.98 ± 1.31^f^	174.16 ± 3.16^a^	89.25 ± 2.39^d^	82.56 ± 1.63^e^	75.95 ± 0.1^f^
Nonane, 2-methyl-	111.23 ± 11.67^b^	40.79 ± 0.03^h^	67.02 ± 2.66 ^g^	46.12 ± 0.75^h^	67.69 ± 1.54^g^	85.04 ± 5.29^def^	92.88 ± 1.93^cd^	86.15 ± 1.71^de^	183.29 ± 2.49^a^	90.94 ± 4.75^cde^	82.45 ± 1.05^ef^	75.31 ± 3.87^fg^
Nonane, 2,5-dimethyl-	106.33 ± 5.39^b^	33.51 ± 2.72^h^	47.25 ± 1.01 ^g^	32.44 ± 1.03^h^	68.04 ± 0.2^f^	82.49 ± 4.05^d^	90 ± 1.25^c^	82.67 ± 3.24^d^	188.04 ± 3.38^a^	92.83 ± 3.43^c^	80.26 ± 0.24^de^	75.39 ± 3.63^e^
N-decane	100.96 ± 2.02^b^	47.92 ± 0.69^f^	64.64 ± 4.34^e^	51.82 ± 0.16^f^	65.04 ± 2.04^e^	81.36 ± 1.65 ^cd^	85.03 ± 1.14^c^	83.44 ± 3.04^cd^	125.75 ± 3.74^a^	100.69 ± 1.22^b^	80.22 ± 1.71^cd^	78.38 ± 1.06^d^
Nonane, 2,5-dimethyl-	109.42 ± 7.19^b^	36.82 ± 0.91^i^	43.77 ± 3.19 ^h^	34.53 ± 1.55^i^	64.5 ± 2.26^g^	84.42 ± 0.88^de^	87.43 ± 1.86^d^	77.86 ± 0.2^ef^	144.38 ± 1.17^a^	99.6 ± 1.8^c^	83.27 ± 1.91^de^	74.75 ± 3.16^f^
Decane, 5-methyl-	106.69 ± 0.95^b^	40.13 ± 0.35^k^	45.31 ± 0.47^j^	37.66 ± 1.4^k^	63.51 ± 3.51^i^	83.93 ± 0.34^f^	88.34 ± 0.54^e^	75.05 ± 0.57^h^	135.02 ± 1.34^a^	93.32 ± 0.96^d^	78.83 ± 1.03^g^	73.35 ± 0.84^h^
Decane, 4-methyl-	115.11 ± 3.47^b^	36.39 ± 1.36^j^	47.19 ± 1.47^i^	43.5 ± 1.82^i^	63.9 ± 2.7^h^	81.68 ± 1.34^e^	87.56 ± 0.27^d^	76.52 ± 1.38^f^	145.05 ± 0.75^a^	97.73 ± 1.71^c^	78.7 ± 1.34^ef^	71.64 ± 0.7^g^
2,6-Dimethyldecane	101.57 ± 0.61^b^	44.94 ± 1.78^g^	40.94 ± 0.51^gh^	39.2 ± 0.84^h^	61.5 ± 3.03^f^	78.21 ± 2.06^e^	83.98 ± 2.29^d^	78.69 ± 1.55^e^	133.85 ± 1.45^a^	96.38 ± 3.04^c^	84.48 ± 1.3^d^	81.39 ± 0.74^de^
Decane, 2,9-dimethyl-	101.44 ± 0.77^b^	45.37 ± 1.62^g^	47.23 ± 3.1 ^g^	38.82 ± 1.06^h^	56.33 ± 0.62^f^	85.03 ± 2.23^c^	83.23 ± 2.42^c^	69.52 ± 1.86^d^	118.09 ± 4.28^a^	84.36 ± 1.47^c^	68.25 ± 1.48^de^	63.66 ± 0.77^e^
N-undecane	85.52 ± 2.06^e^	75.13 ± 1.97^f^	68.49 ± 1.72^f^	61.16 ± 3.21^g^	74.86 ± 2.44^f^	105.43 ± 1.23^ab^	98.27 ± 1.89^cd^	95.09 ± 0.22^d^	103.73 ± 1.59^abc^	107.12 ± 7.49^a^	98.5 ± 3.45^bcd^	96.59 ± 1.74^d^
Decane, 3,7-dimethyl-	79.91 ± 3.37 ^fg^	60.28 ± 3.76^i^	52.01 ± 0.47^j^	50.41 ± 1.69^j^	64.22 ± 3.39^i^	94.53 ± 1.23^d^	82.22 ± 0.38^f^	116.18 ± 1.57^b^	122.85 ± 1.4^a^	89.03 ± 1.12^e^	77.01 ± 0.42^g^	70.9 ± 2.78^h^
Decane, 2,9-dimethyl-	84.08 ± 3.05^e^	59.73 ± 4.62^gh^	54.41 ± 0.49^hi^	52.18 ± 1.13^i^	66.03 ± 3.81^g^	93.86 ± 0.31^bc^	85.93 ± 3.07^de^	83.24 ± 5.27^e^	116.8 ± 1.75^a^	92.31 ± 0.32^cd^	81.22 ± 0.01^e^	74.3 ± 0.7^f^
N-dodecane	90.88 ± 0.25 ^cd^	81.59 ± 0.28^e^	60.75 ± 0.69 ^g^	62.66 ± 2.21^g^	72.21 ± 1.24^f^	94.55 ± 1.54^bc^	94.73 ± 1.61^bc^	107.96 ± 3.56^a^	107.99 ± 5.93^a^	108.9 ± 1.89^a^	91.95 ± 2.76^c^	85.36 ± 1.36^de^
Undecane, 2,5-dimethyl-	90.74 ± 2.45^c^	74.08 ± 2.79^d^	53.91 ± 0.12^f^	57.2 ± 1.98^ef^	66.19 ± 1.12^de^	92.57 ± 2.89^c^	93.99 ± 3.79^c^	111.34 ± 12.57^b^	137.39 ± 4.24^a^	111.57 ± 1.83^b^	92.73 ± 3.2^c^	92.27 ± 0.39^c^
Dodecane, 4-methyl-	87.68 ± 0.8^bcd^	62.56 ± 9.48^cde^	42.79 ± 11.66^e^	56.27 ± 3.47^de^	58.68 ± 7.67^cde^	93.52 ± 31.38^bc^	90.32 ± 6.21^bcd^	92.2 ± 19.57^bcd^	142.96 ± 33.67^a^	116.15 ± 1.53^ab^	89.68 ± 3.24^bcd^	88.94 ± 11.49^bcd^
2,6,10-Trimethyltridecane	85.36 ± 9.4^bc^	74.42 ± 12.77^cd^	42.03 ± 14.8^d^	66.26 ± 9.39^cd^	67.94 ± 7.4^cd^	89.13 ± 32.39^bc^	90.65 ± 2.62^bc^	94.15 ± 24.98^abc^	128.9 ± 20.6^a^	114.34 ± 3.99^ab^	99.09 ± 2.4^abc^	91.58 ± 16.49^abc^
Dodecane, 4-methyl-	87.37 ± 15.4^ab^	80.4 ± 16.34^ab^	41.33 ± 14.42^c^	64.74 ± 9.12^bc^	69.39 ± 4.65^abc^	92.96 ± 36.96^ab^	93.82 ± 6.7^ab^	80.47 ± 10.56^ab^	103.22 ± 3.13^a^	98.79 ± 0.53^ab^	89.68 ± 7.88^ab^	83.58 ± 15.1^ab^
Dodecane, 4,6-dimethyl-	97.55 ± 3.29 ^cd^	76.07 ± 1.22^e^	51.05 ± 2.02^f^	59.84 ± 5.18^f^	69.57 ± 3.96^e^	89.79 ± 1.3^d^	93.21 ± 3.05^cd^	117.18 ± 6.41^a^	115.55 ± 7.76^a^	107.83 ± 0.13^ab^	96.63 ± 4.52^cd^	90.69 ± 2.34^cd^
Dodecane, 2,7,10-trimethyl-	103.57 ± 5.05^c^	98.86 ± 1.1^cd^	60.87 ± 5.56^g^	70.15 ± 1.28^f^	72.08 ± 0.82^f^	89.83 ± 2.41^e^	94.68 ± 2.86^de^	128.56 ± 4.22^a^	113.78 ± 6.15^b^	114.78 ± 2.6^b^	99.11 ± 3.86^cd^	92.29 ± 3.49^de^
N-tridecane	110.86 ± 1.92^bcd^	106.65 ± 4.28^cde^	73.42 ± 1.11^g^	74.79 ± 1.67 ^g^	88.17 ± 2.09^f^	101.89 ± 3.47^de^	113.08 ± 8.12^bc^	133.23 ± 6.06^a^	117.31 ± 2.8^b^	106.18 ± 2.71^cde^	107.97 ± 8.03^bcde^	100 ± 0.91^e^
Tetradecane, 5-methyl-	103.73 ± 5.88^bc^	99.09 ± 0.38^c^	51.42 ± 0.62^g^	64.06 ± 1.05^f^	74.15 ± 0.28^e^	90.27 ± 0.97^d^	98.97 ± 0.93^c^	115.55 ± 2.47^a^	110.54 ± 3.85^ab^	104.89 ± 1.28^bc^	102.75 ± 8.05^c^	90.56 ± 1.13^d^
Dodecane, 2,6,11-trimethyl-	97.36 ± 1.04^bc^	103.49 ± 4.52^b^	50.41 ± 1.16^h^	68.63 ± 3.86^g^	72.53 ± 0.27^fg^	80.23 ± 2.44^ef^	93.25 ± 2.78 ^cd^	118.78 ± 11.39^a^	102.1 ± 0.04^bc^	96.91 ± 1.8^bc^	94.68 ± 1.57^bcd^	87.52 ± 0.85^de^
N-tetradecane	107.66 ± 1.74^a^	100.57 ± 2.9^ab^	65.79 ± 3.54^e^	85.11 ± 0.49^d^	103.09 ± 7.85^ab^	105 ± 7.6^ab^	88.39 ± 0.38^cd^	103.72 ± 4.23^ab^	100.39 ± 2.64^ab^	96.39 ± 1.66^bc^	100.4 ± 2.61^ab^	87.26 ± 3.19^cd^
Pentadecane	121.73 ± 6.42^ab^	103.65 ± 0.11^cde^	76.96 ± 0.3 ^g^	119.65 ± 0.82^abc^	127.37 ± 9.55^a^	119.69 ± 12.02^abc^	105.72 ± 2.48^bcde^	113.74 ± 16.18^abcd^	114.6 ± 0.17^abcd^	93.41 ± 1.09^efg^	127.07 ± 8.48^a^	84.32 ± 0.48^fg^
Total amount	97.16 ± 0.02^bc^	66.11 ± 1.04^i^	55.55 ± 1.71^j^	55.92 ± 0.38^j^	71.14 ± 0.28^h^	89.36 ± 1.98^ef^	89.18 ± 0.15^ef^	91.15 ± 1.4^de^	125.18 ± 3.97^a^	94.52 ± 0.71^cd^	85.91 ± 1.38^f^	78 ± 1.63^g^
*Ester*												
Oxalic acid, 2-ethylhexyl pentyl ester	107.28 ± 0.65^b^	44.51 ± 1.03^g^	40.31 ± 3.88^g^	39.26 ± 2.03^g^	60.02 ± 3.91^f^	83.37 ± 2.09^d^	87.67 ± 0.65^d^	72.57 ± 3.03^e^	139.7 ± 3.49^a^	98.22 ± 1.36^c^	76.21 ± 3.23^e^	76.45 ± 1.48^e^
Acetic acid, 2-ethylhexyl ester	124.43 ± 2.63^a^	97.73 ± 1.84^b^	60.83 ± 2.62^ef^	73.61 ± 3.22^d^	65.4 ± 1.91^e^	88.84 ± 0.97^c^	64.8 ± 2.17^e^	58.28 ± 2.86^f^	77.08 ± 1.71^d^	65.41 ± 3.53^e^	65.1 ± 0.54^e^	52.62 ± 0.95^g^
Carbonic acid, undecyl vinyl ester	98.41 ± 5.03^b^	68.77 ± 0.42^cde^	47.88 ± 0.09^f^	55.86 ± 1.07^ef^	62.09 ± 3.31^de^	68.28 ± 1.53^cde^	71.44 ± 2.46^cd^	56.15 ± 2.48^ef^	116.15 ± 17.08^a^	96.3 ± 4.46^b^	77.96 ± 2.48^c^	68.77 ± 0.23^cde^
Sulfurous acid, hexyl pentadecyl ester	90.63 ± 0.82^bc^	87.85 ± 1.02^bc^	52.89 ± 0.6^f^	58.45 ± 1.17^ef^	56.98 ± 1.5^ef^	53.65 ± 3.59^ef^	80.88 ± 1.42^cd^	62.08 ± 0.04^ef^	117.98 ± 23.51^a^	103.07 ± 2.13^ab^	79.07 ± 0.26 ^cd^	69.82 ± 2.52^de^
Tributyl phosphate	181.32 ± 18.2^d^	258.85 ± 0.64^c^	337.93 ± 17.56^b^	420.31 ± 0.73^a^	104.59 ± 2.88^e^	103.3 ± 5.13^e^	85.1 ± 3.68^fg^	63.1 ± 2.39^h^	83.76 ± 1.6^fg^	67.86 ± 0.29^gh^	53.12 ± 0.14^hi^	40.55 ± 0.91^i^
Total amount	120.41 ± 1.81^b^	111.54 ± 0.15^c^	107.97 ± 3.12^c^	129.5 ± 0.83^a^	69.81 ± 0.23 ^g^	79.49 ± 0.39^f^	77.98 ± 0.83^f^	62.43 ± 0.21^h^	106.93 ± 7.44^c^	86.17 ± 0.57^e^	70.29 ± 0.28^g^	61.64 ± 0.17^h^
Aromatic												
Ethylbenzene	101.82 ± 3.61^a^	67.6 ± 0.39 ^g^	70.53 ± 1.92^fg^	51.78 ± 2.81^h^	75.03 ± 2.31^def^	99.96 ± 0^a^	79.63 ± 2.67^cd^	80.63 ± 0.85^c^	93.64 ± 4.29^b^	76.78 ± 1.65^cde^	76.64 ± 0.98^cde^	73.43 ± 1.67^ef^
Mesitylene	104.1 ± 1.02^a^	87.06 ± 2.39^bc^	74.69 ± 0.92^d^	60.47 ± 1.21^e^	76.33 ± 3.63^d^	76.04 ± 2.55^d^	78.38 ± 0.49^cd^	79.73 ± 4.15^cd^	89.71 ± 7.35^b^	76.73 ± 8.11^d^	78.06 ± 3.5^cd^	73.41 ± 3.47^d^
Benzene, 1,3-bis(1,1-dimethylethyl)-	102.56 ± 4.01^abc^	35.57 ± 1.4^e^	35.38 ± 0.17^e^	30 ± 1.94^e^	102.77 ± 2.62^ab^	103.65 ± 7.97^a^	106.86 ± 11.26^a^	103.72 ± 3.99^a^	106.38 ± 2.38^a^	92.48 ± 0.34^bcd^	88.82 ± 2.15^d^	91.87 ± 2.65^cd^
Total amount	102.83 ± 0.21^a^	63.41 ± 0.46 ^g^	60.2 ± 1^g^	47.42 ± 1.18^h^	84.71 ± 1.31^ef^	93.21 ± 3.5^cd^	88.29 ± 4.81^de^	88.03 ± 2.43^de^	96.58 ± 0.23^bc^	82 ± 3.37^f^	81.17 ± 1.56^f^	79.57 ± 1.48^f^

### 3.9 Effect of PAW combined with US on the microstructure of silkworm pupae

**Fig. 4:**
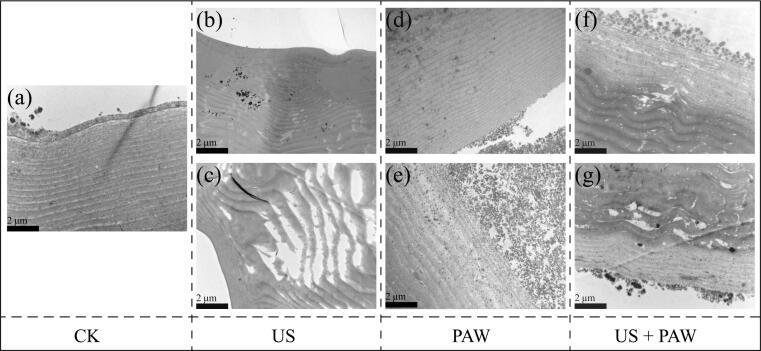
The microstructure of silkworm pupae epidermis at 12,000x under US, PAW and PAW combined with US treatment, CK (a), US treatment for 20 min (b) and 40 min (c), PAW treatment for 20 min (d) and 40 min (e), PAW combined with US treatment for 20 min (f) and 40 min (g).

As depicted in Fig. 4, the epidermis of fresh silkworm pupae, featuring a chitin-protein complex, exhibited a layered structure. Following 40 min of US treatment, this layered structure was disrupted, revealing conspicuous gaps. Ultrasonic cavitation in liquid media alternately compresses and stretches the molecular structure of the medium, leading to fluid rupture and the formation of microbubbles. The high pressure resulting from the collapse of microbubbles can disrupt the chitin-protein complex. It has been reported that US disrupts non-covalent bonds between protein chains, thereby altering the tertiary structure of proteins and subsequently influencing their microstructure [11], [38]. Moreover, upon the combination of PAW and US, impurities became apparent on the outer surface of the silkworm pupal epidermis due to PAW infiltration. Since the epidermis of silkworm pupae contains active substances, such as proteins, fats, amino acids, and phenols, the free radicals in PAW interact with these substances. The generated products flow out through the cracks under the influence of US. Research has indicated that the formation of cavitation bubbles and microjets by ultrasound is associated with increased temperature and pressure. This disturbance not only affects the cell membranes of the substance, enabling the discharge of constituents into the immediate environment, but also facilitates the penetration of PAW into the material [39]. Therefore, as the duration of US combined with PAW treatment increased, the layered structure was further disrupted. This demonstrates that the combination of US and PAW can break the epidermal layered structure of silkworm pupae, promote PAW penetration, and induce irreversible effects on the proteins, fats, enzymes, amino acids, and phenols of silkworm pupae. Additionally, Chemat et al. reported that US can promote the formation of small temporary channels in the plasma membrane of cells, through which PAW can enter cells or food for effects [39].

### 3.10 Effect of PAW combined with US on the microbial diversity of silkworm pupae

The fresh silkworm pupae are from the cocoon does not contains any microorganism. But, the microorganism in silkworm pupae is far different with the storage environment. The impact of PAW combined with US treatment on the microbial diversity of silkworm pupae is illustrated in Fig. 2 (e), showcasing taxa such as *Streptococcaceae, Leuconostocaceae*, *Acetobacteraceae*, and *Caulobacteraceae*. Differential shifts in relative abundance underscore their susceptibility to the combined treatment. It is noteworthy that while the relative abundances of *Caulobacteraceae* and *Oxalobacteraceae* exhibited minor changes, there were significant declines of 5 %, 7 %, and 5 % in the levels of *Streptococcaceae*, *Leuconostocaceae*, and *Acetobacteraceae*, respectively. The enhanced penetration of reactive species generated by PAW into bacterial cells, facilitated by US-induced agitation, can be attributed to the synergistic effect of US and PAW. US induces acoustic cavitation, disrupting cell wall structure, increasing cell membrane permeability, thinning membranes, and creating localised areas of high temperature and pressure, thereby incapacitating microorganisms [9]. In contrast, PAW contains RONS, inducing oxidative stress on bacterial cell membranes, resulting in membrane disruption, organelle and protein damage, nucleic acid impairment, and finally cell death [17]. These findings align with our study in terms of antioxidative properties, microstructural characteristics, and enzymatic activities. Furthermore, another study demonstrated that after 40 min of combined treatment with US and PAW, the total viable bacterial count (TVC) of crayfish was reduced by 1.17 log CFU/g. The decrease in bacterial count is attributed to acidic species and RONS within the PAW, with the potential to cause oxidative and nitrosative damage to microorganisms by inducing DNA destruction, lipid peroxidation, and inhibiting enzyme activity [40]. Additionally, Qian et al. noted that a myofibrillar gel prepared with PAW demonstrated antibacterial properties against *Salmonella enteritidis* and *Staphylococcus aureus* due to the presence of RONS, meanwhile the pH characteristic of PAW allowed sterilization to occur [7]. In conclusion, this combination of technologies holds promise as an effective and eco-friendly sterilization method across various fields, including food safety and medical device sterilization, contingent upon the identification of appropriate parameters.

## 4 Conclusion

A nonthermal treatment method for PAW combined with US is proposed in this study. The results demonstrate a significant improvement in the quality of silkworm pupae following treatments with PAW and US. The reduction in the relative abundance of microorganisms in the silkworm pupae is accompanied by a disruption in the microstructure after PAW combined with US treatment. This disruption leads to the formation of small temporary channels that enhance the availability of unsaturated fatty acids, amino acids, flavour components, and phenols. Ultimately, this process enhances the antioxidant capacity and nutritional value of the silkworm pupae. However, prolonged PAW combined with US treatment not only modifies the protein and enzyme structures within silkworm pupae but also induces the oxidation of unsaturated fatty acids and amino acids due to the high activity of PAW. This results in the generation of MDA, although PAW may neutralise them under acidic conditions. Therefore, optimizing the processing conditions is imperative to maximise the quality of silkworm pupae, enabling a broader application of this non-thermal processing technology within the industry.

## CRediT authorship contribution statement

**Jia-Bao Ni:** Writing – original draft, Software, Methodology, Conceptualization. **Shi-Ye Luo:** Writing – review & editing, Software. **Yan-Xiang Bi:** Writing – review & editing, Conceptualization. **Sara Zielinska:** Writing – review & editing. **Chang-Jiang Ding:** Writing – review & editing, Formal analysis. **Jia-Li Tao:** Writing – review & editing. **Zhen Ning:** Software. **Wen-Li Tian:** Funding acquisition, Conceptualization. **Wen-Jun Peng:** Funding acquisition, Conceptualization. **Xiao-Ming Fang:** Writing – review & editing, Supervision, Resources, Conceptualization.

## Declaration of competing interest

The authors declare that they have no known competing financial interests or personal relationships that could have appeared to influence the work reported in this paper.
